# Comparative Evaluation of Different Numerical Pain Scales Used for Pain Estimation during Debonding of Orthodontic Brackets

**DOI:** 10.1155/2021/6625126

**Published:** 2021-03-04

**Authors:** Mohmed Isaqali Karobari, Ali A Assiry, Mubashir Baig Mirza, Fazlur Rahman Sayed, Sufiyan Shaik, Anand Marya, Adith Venugopal, Mohammad Khursheed Alam, Rithvitou Horn

**Affiliations:** ^1^Conservative Unit, School of Dental Sciences, Universiti Sains Malaysia, Health Campus, Kubang Kerian 16150, Kota Bharu, Kelantan, Malaysia; ^2^Preventive Dental Science Department, Faculty of Dentistry, Najran University, Najran, Saudi Arabia; ^3^Conservative Dental Science Department, College of Dentistry, Prince Sattam bin Abdulaziz University, AlKharj, Saudi Arabia; ^4^Badr Al Samaa Group of Hospitals, Muscat, Oman; ^5^Happy Mouth Dental Clinic, Mumbai, Maharashtra, India; ^6^Department of Orthodontics, University of Puthisastra, Phnom Penh, Cambodia; ^7^Orthodontic Division, Preventive Dentistry Department, Jouf University, Sakakah, Saudi Arabia; ^8^Faculty of Dentistry, University of Puthisastra, Phnom Penh, Cambodia

## Abstract

**Introduction:**

Patients experience various levels of discomfort during orthodontic treatment, i.e., after placement of separators, orthodontic implant placement, and archwire placement and during debonding. Various pain control methods have been developed to relive pain during debonding, i.e., finger pressure (FP), elastomeric wafer (EW), and stress relief (SR).

**Aim:**

To analyse various pain scales commonly used to determine the effect of different pain control methods during debonding of orthodontic brackets. *Study Design*. A comparative cross-sectional study performed on a sample of 60 patients (*n* = 60) including 14 males and 46 females who were ready for debonding and who were divided into three groups, i.e., finger pressure (FP), elastomeric wafer (EW), and stress relief (SR).

**Materials and Methods:**

A 100 mm Visual Analog Scale (VAS) was used to record the pain intensity for each tooth. Another scale known as Pain Catastrophizing Scale (PCS) was used to evaluate the patient's general attitude towards pain perception. The armamentarium and operator were kept same for all the patients. Statistical analysis used was the Kruskal–Wallis test, used for intergroup and intragroup comparison of pain scores.

**Results:**

Lowest total pain score was recorded in the FP group (*P*=0.043) on intergroup comparison, while on intragroup comparison, higher pain scores were recorded in lower anterior region (*P*=0.02) in all three groups. There was no significant difference between the pain scores reported by the male and female subjects.

**Conclusion:**

FP is an effective method of pain control. And teeth in the anterior region of lower and upper arches are more sensitive to pain. In terms of cognitive-affective constructs, although the VAS has been widely used in previous studies, the PCS has been detailed to show the most reliable association with physical discomfort and emotional distress.

## 1. Introduction

Even in light of all recent developments in dentistry, the most common complaint of many patients is that of pain or discomfort after various types of dental treatments which include orthodontic therapy as well [1, 2]. Pain is a subjective experience which varies amongst every patient and is expressed by them in varying degrees, during the phase of active treatment and also during the removal of the fixed appliance [1, 3]. Pain is ranked first amongst least liked parameters during treatment and fourth amongst all fears and anxiety prior to orthodontic treatment as per a survey conducted in 2000 [4, 5]. Among all patients undergoing orthodontic treatment, almost 70–95% have reported varying degrees of pain during orthodontic treatment which has been the reason for them to discontinue orthodontic treatment [6–12]. Patients experience varying degrees of discomfort in different clinical situations, i.e., after placement of separators, orthodontic implant placement, archwire placement and activations, banding, and elastic wear and while debonding which are often expressed by them as feelings of pressure, tension, soreness of the teeth, and pain as such [6]. These perceptions may be due to changes in blood flow in the periodontal ligament and correlated with the presence of prostaglandins, neuropeptides like substance P, cytokines, and other inflammatory mediators [10, 13–15].

Pain at debonding was first studied in depth by Williams and Bishara [3] who concluded that patients could withstand intrusive forces the most. Normando et al. in their study assessed the degree of pain during debonding with two instruments and concluded that the lift-off instrument lowered the pain levels twice that by wire cutting pliers [3]. Another study conducted by Mangnall et al. evaluated the effect of soft acrylic bite wafers and found significantly lesser pain in the posterior region compared to the anterior region [16]. Conclusions of the previous studies have drawn the attention of researchers towards the direction of methods to control effects of anatomic location and personal differences in pain experience during debonding. Thus the aim of the study was to determine the effect of different pain control methods on the pain perception by the patient during debonding of orthodontic brackets. And the objectives were to compare the efficacy of two different pain scales for evaluation of three different pain control methods used for debonding.

## 2. Materials and Methods

The study was carried out with approval from institutional ethical committee at Faculty of Dentistry, Najran University, with the assigned ethical approval number (2020/00116). A sample of 60 (*n* = 60) subjects including 14 males and 46 females obtained from the OPD were divided into 3 groups, finger pressure (FP) group, elastomeric wafer (EW) group, and stress relief (SR) group (Table 1); all subjects in their finishing stage of treatment and ready for debonding were evaluated for pain perception at the day of debonding and one week before debonding. The sample size was determined using a computer program (Minitab version 18, Minitab Inc., State College, Pennsylvania, USA). It was seen that, on including 20 subjects per group, there would be a power of 80% for a clinically significant difference. The sample size estimation was computed using a significance level of 0.05 and power of 80%. It has been seen previously that a minimum mean change of 13 mm has been proved to have a clinically significant impact across the Visual Analog Scale [17]. Studies conducted using the Pain Catastrophizing Scale have shown that there needs to be a minimum of 50 subjects for assessment of reproducibility and construct validity [18].

The inclusion criteria for the selection of subjects included patients in finishing stage of treatment, ready to be debonded, aged between 13 and 24 years, and who could understand, assess, and answer the questionnaires; patients undergoing fixed orthodontic treatment for upper and lower arch with Ormco Mini 2000 MBT prescription 0.022-inch metal brackets (Ormco, Glendora, California, USA) with a single-mesh base and bonded with 3M Transbond XT light cure adhesive (3M Unitek Orthodontic Products, Monrovia, California, USA), and with 0.019” X 0.025” stainless steel wire (3M Unitek, Monrovia California, USA) placed in both arches with absence of any loose bracket; patients with no history of medicine intake periodically or in the last 24 hours, particularly pain modifying drugs (analgesics, anti-inflammatory, anxiolytic drugs, etc.).

On the other hand, the exclusion criteria for the subjects included patients with any missing teeth except extracted premolars or any prosthesis, heavily restored teeth, and root canal treated teeth; patients with history of any previous surgical treatment (including impacted tooth eruption) and any craniofacial deformities that would affect dentoalveolar bone quality (e.g., cleft lip and palate). Patients with any active periodontal problem (recession and mobility greater than Grade I) were also excluded.

The armamentarium for debonding the brackets was common to all the patients, i.e., one operator with the same debonding pliers but using three different pain control methods. Eltee debonding pliers curved DD-009, a short lever arm curved type (Libral traders, New Delhi, India), were used for all the subjects.

Patients were randomly divided into different groups as follows:

- FP group: during debonding of each bracket, the operator's finger pressure was applied from the incisal or occlusal surface of the tooth in a gingival direction with the thumb. In order to remove the influence of the occlusal morphological variations, a cotton roll was held under a thumb.
- EW group: the heavy-body silicone printing impression material, which was about 5-6 mm thick, made an arch-formed bite raiser named as wafer bite [19]. It was positioned between the arches, and during bracket removal patients were asked to bite it firmly. It was sterilized 10 minutes before use by immersing it in 2% active gluteraldehyde solution [20].
- SR group: the method was supplemented with regular debonding. Patients were told not to occlude their teeth when brackets were being taken off. The patient stress was relieved by telling them that in debonding no serious discomfort or pain will occur. The method is based on cognitive behavior therapy that is primarily directed against the psychological mechanism of pain in patients [21].

**Figure 1:**
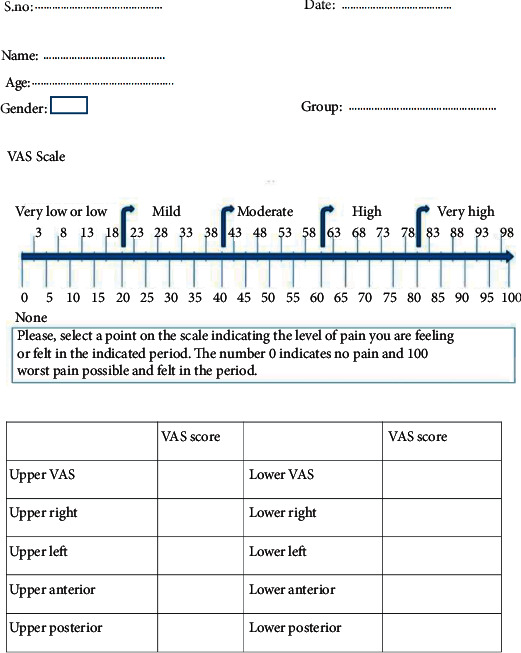
The form used to collect data using the Visual Analog Scale.

Patients who wanted to participate in the study were asked to fill in the consent form and were provided a Visual Analog Scale (VAS) questionnaire form (Figure 1) for recording the intensity of pain felt [21]. This questionnaire contained a 100 mm scale with 0 on one end and 100 on the other prepared for each tooth, wherein score 0 means “no pain,” 100 means “worst pain,” and increasing scores from 0 to 100 represents pain increase. All of the patients were interviewed by the same operator who performed the debonding, beaks of the pliers were engaged occlusogingivally between the bracket base and adhesive, and a gentle torquing movement was applied for debonding the brackets and the archwires were not removed before debonding. Brackets on incisors, canines, and premolars were debonded one at a time, starting from upper right to upper left quadrant followed by lower right to lower left quadrant.

For recording the scores, patients were asked to put a vertical mark on the scale according to the intensity of pain felt by them after each bracket was debonded. The distance from the low end of the scale to the patient's mark was measured using a metallic scale to obtain a score for the particular tooth. Based on the scores obtained for all the teeth, total VAS and other subscores for different locations in arch were calculated by the operator. To prevent operator bias, the VAS scores were evaluated by another operator who was blinded to the groups.

**Figure 2:**
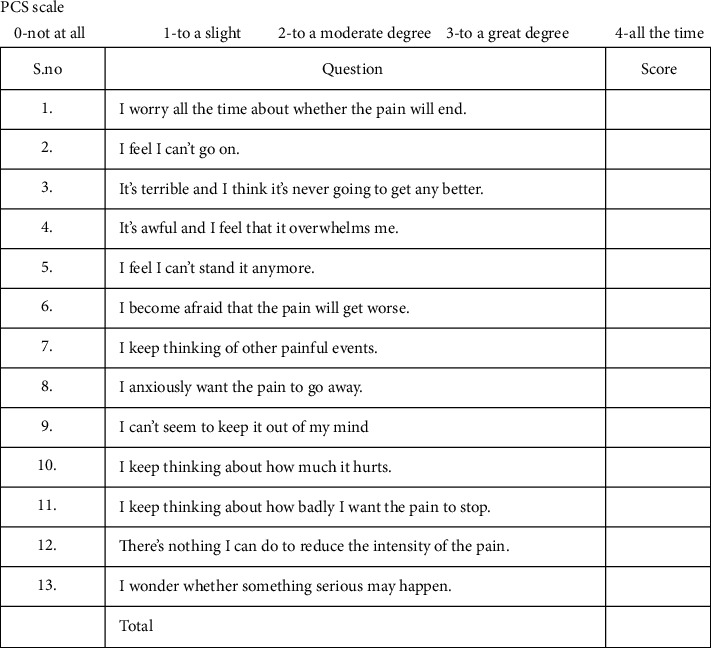
The form used to collect data using the Pain Catastrophizing scale.

Patients were asked to complete another questionnaire form containing the Pain Catastrophizing Scale (PCS) (Figure 2), which helped to evaluate the correlation between personality traits and actual pain during debonding. The PCS form contained 13 different statements, each detailing different kinds of pain sensations. Each patient was asked to use a 5-point scale and rate each statement in terms of pain severity ranging from 0 to 4. To circumvent connections of their actual pain experience and general response to painful situations which would have affected their PCS score, patients were asked to complete the questionnaires one week before debonding. The scores were recorded as total PCS score and individual scores of three subscales: rumination, magnification, and helplessness.

## 3. Statistical Analysis Plan

The descriptive statistics about the patient distribution into different groups along with age and sex and other processed data including PCS scores for different pain control methods, effect of sex on VAS and PCS score, correlations of subscales, and total scores of PCS with VAS scores have been presented in form of tables and graphs. With the help of IBM SPSS 25.0.0.0 (IBM Corp., Armonk, NY, USA), Kruskal–Wallis test was carried out for finding any statistically significant difference in the pain score for different pain control method, for intragroup evaluation (i.e., same pain control method for different quadrants), and for determining the effect of catastrophizing on the pain score for different groups.

Linear regression analysis was performed directly in Excel using the XLSTAT (Addinsoft Inc., USA) add-on software to find correlation between the VAS score and the pain control methods with score adjusted and not adjusted for age and sex.

## 4. Results

The matching between the groups is shown in Table 1. A total of 60 subjects included 14 males with mean age of (19.85 ± 2.03) and 46 females with mean age of (19.43 ± 2.88) and the mean age for group 1 was (18.80 ± 2.37), group 2 (19.90 ± 3.19), and group 3 (19.90 ± 2.44). A Kruskal–Wallis test showed mean value for different subgroups and the total PCS. On conducting the post hoc analysis, it was seen that there were no significant differences between the male and female groups.

**Figure 3:**
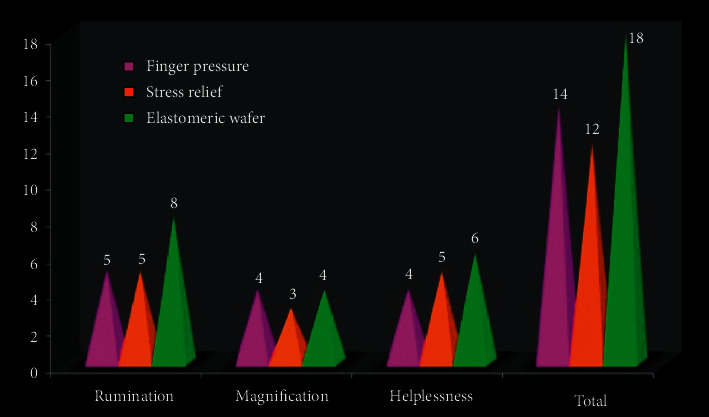
Median score for PCS.

Statistically significant differences in pain score with PCS and different subscales between the different drug treatments were seen, i.e., rumination *χ*^2^ = 18. 20, *P* ≤ 0.001, magnification *χ*2 = 10. 40, *P*=0.02, helplessness *χ*2 = 18. 20, *P* ≤ 0.001, and total = 19.39, *P* ≤ 0.001. The PCS score was compared between the groups and showed statistically significant difference between the groups (Table 2; Figure 3).

**Figure 4:**
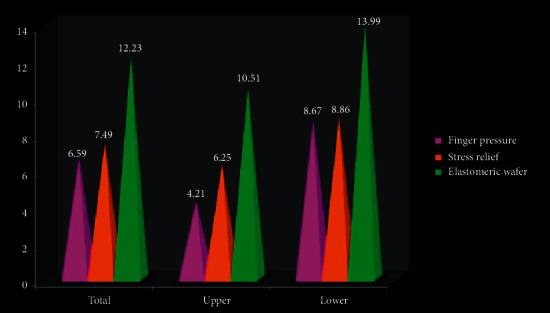
Median score for VAS.

On intergroup comparison, the results showed a statistically significant difference in the total VAS score between groups with lowest total pain score recorded in FP group with *P*=0.043. Kruskal–Wallis test was used to find the difference in VAS pain scale and different subscale among different treatment groups. The mean value for different subgroups and total VAS is shown in Table 2. The result was of statistically significant difference in pain score with VAS and different subscale between the different drug treatments (Tables 3 and 4; Figure 4).

**Figure 5:**
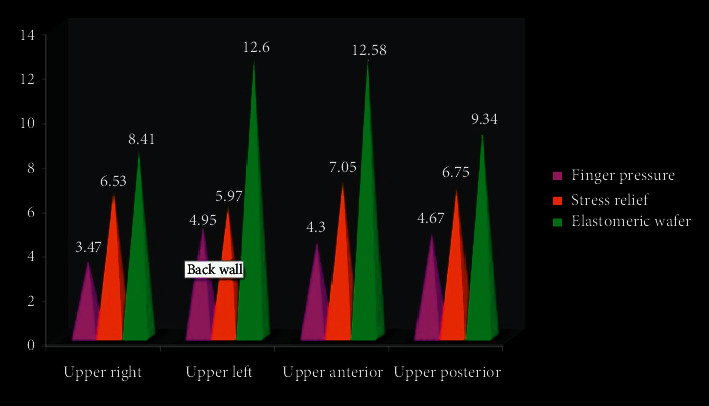
Median score for VAS for upper quadrant.

On intragroup comparison to evaluate the effect of location, a statistically significant difference in VAS score was obtained in all three groups and consistent higher pain scores were recorded in lower anterior region with a *P*=0.02, in all three groups. In the upper jaw, statistical difference in VAS between the different drug treatments was calculated. Total average median score in the upper right posterior quadrant for finger pressure was found to be 3.47, stress relief was 6.53, and elastomeric wafer was 8.41 with *χ*^2^ = 5.08, *P*=0.08 and was not statistically significant. On the upper right posterior quadrant, average median score for VAS was statistically significant and detailed as 4.95 for finger pressure, 5.97 for stress relief, and 12.60 for elastomeric wafer with *χ*^2^ = 9.03, *P*=0.037 (Table 3; Figure 5).

**Figure 6:**
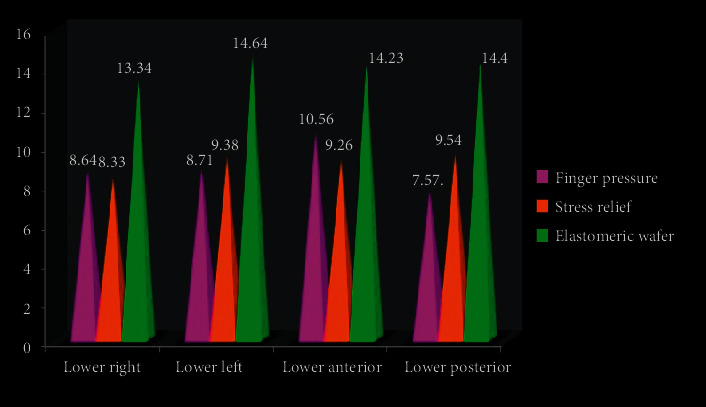
Median score for VAS for Lower quadrant.

In the lower jaw, statistically significant differences in VAS between the different drug treatments were calculated. The total average median score in the lower right posterior quadrant for finger pressure was 8.64, stress relief was 8.33, and elastomeric wafer was 13.34 with *χ*^2^ = 2.8, *P*=0.223 and was not statistically significant. On the lower left posterior quadrant, average median score for VAS was statistically significant and detailed as 8.71 for finger pressure, 9.38 for stress relief, and 14.64 for elastomeric wafer with *χ*^2^ = 9.03, *P*=0.037 (Table 3; Figure 6).

## 5. Discussion

This study was designed to evaluate the efficacy of different pain control methods which can be used for debonding of orthodontic brackets with minimal discomfort and other important determinants of pain such as general attitude or thoughts of patient and location of the tooth. The effect of other determinants such as age and gender of the patients were also observed. Results of intergroup evaluation showed that the patients in FP group perceived lesser pain during debonding than the patients in SR and EW group. So it can be said that FP method was a better method of pain control when compared to the SR and EW method.

Taking into consideration the location of teeth, the results of our study showed that maximum pain scores were recorded in the lower anterior region of jaw followed by upper anterior region of jaw, whereas least pain scores were recorded in upper posterior and lower posterior regions irrespective of the group.

On comparing the different pain control methods with respect to different locations of teeth, it was found that, except for the upper right quadrant, FP was an effective method for pain control in both upper and lower arches when compared to EW and SR method. A similar study was conducted by Nehir et al. to ascertain the pain experience during bracket removal and the consequences of different methods of controlling pain, sex, and personal traits on the pain experience and they reported that FP method was more efficacious than the EW and SR method, especially in the lower jaw [19]. An important observation of our study agreed with previous literature about the impact of sex on pain perception; i.e., higher VAS scores were recorded for females [6, 7, 10, 13]. Age was also reported to be a crucial factor in pain perception in previous studied [19]. Therefore, we formulated the inclusion criteria of a limited range of age and made adjustments for age during statistical analysis to eliminate its effects on the final score (Tables 5 and 6).

Since pain catastrophizing has been an anticipator of pain perception among different age groups, sex and patient's general attitude could be critical during recording of scores [22–25]. Thus it was decided to use Pain Catastrophizing Scale developed by Sullivan et al. in this study [26]. Patients were requested to answer the PCS questionnaire one week before debonding to avoid any connections with their ratings on PCS and their actual pain experience. It was observed that mean of overall VAS for debonding procedure was less than the values reported for other orthodontic operations such as for separator placement and initial archwire placement [10, 27–31]. In this study, the median VAS total scores of different groups were between 6.59 and 12.23. This variation in pain perception could be due to biological pathways to compensate pain but it could also be the result of “getting used to it” because of the past experiences stored in the brain [20].

In this study, FP method which was designed to determine the effect of intrusive forces was found to be more efficacious than SR and EW in reducing pain based on lesser overall VAS score, upper total score, and lower total score except for the upper right quadrant score which reveals the effectiveness of intrusive force applied on incisal or occlusal surface of the tooth during debonding.

Very less is known about how much of a decrease in pain score for any method can be considered as “clinically successful” for orthodontic applications. Todd et al. explained that a method allowing a reduction of 13 mm on a 100 mm VAS can be agreed as clinically significant [17]. Considering this theory, none of the pain control methods used in our study can be considered as efficient. Still, FP can be considered as an easy and effective technique of pain control, particularly in the lower and upper anterior region in comparison SR and EW method, since it is inexpensive, less time consuming, and less technique sensitive.

On the other hand, the SR method should also be considered as an effective technique by orthodontists as it is known that patients who trust their doctors are more comfortable during orthodontic procedures [19]. It can be suggested to use FP and SR as a combination to effectively control the pain and discomfort during bracket removal.

The anatomic location of teeth and their root morphology can be held responsible for variation in pain experience in different quadrants of arch. Mangnall et al. in their study found that 39% of the patients reported maximum pain in the mandibular anterior region during debonding [16].

In the present study, PCS total and rumination subscale scores were significantly correlated with VAS scores for all the locations except for the upper right quadrant, where debonding started. Lesser VAS score in upper right quadrant from where the debonding starts can be explained as the tendency to underreport the pain felt at the beginning of the procedure or it may also be due to a more comfortable working position for the orthodontist on patient's right side which facilitates an easy accessibility and placement of debonding pliers and efficient debonding. Contrary to our finding, Nehir et al. in their study found lesser VAS score in the lower arches away from where debonding started and explained this correlation with “monotony factor” [19], which means that patient can lose interest in the procedure after the first few teeth are debonded [3].

One finding that has been previously reported in a similar context is that orthodontic debonding usually leads to enhanced tooth sensitivity mostly in the anterior region but it gradually subsides in the next few days [32]. To this effect, desensitizing agents can be applied to help reduce pain during the period after debonding [33].

There have been similar studies conducted across other regions involving various debonding methods in which anxiety scores across different genders have not turned out significantly different [34]. The patients' attitude towards pain depends on varied conditions such as using different hand instruments at debonding, cultural background, intake of analgesics, the periodontal condition of teeth, practitioner's experience, and position of patient and practitioner; the relation of all these parameters to pain perception can be the matter of further study.

## 6. Limitations

Few of the limitations associated with the present study include use of a similar Bite Wafer for all the patients in group, instead of which individualized Bite Wafers could have been used for a better adaptation and proper intrusive force application while biting. Split mouth technique could have been a better method for intergroup comparison. As far as analyzing the results based on the use of scales was concerned, it has been shown in previous studies that the Pain Catastrophizing Scale is immensely useful in case of patients that show heightened distress responses whenever they undergo medical treatment [21]. Both the Visual Analog Scale and the Pain Catastrophizing Scale are reliable and valid methods when it comes to estimating the intensity of physical and emotional discomfort of a patient undergoing clinical procedures. In terms of cognitive-affective constructs, although the VAS has been widely used in previous studies, the PCS has been detailed to show the most reliable association with physical discomfort and emotional distress [21]. In addition, some patients may already be using desensitizing pastes which could cause an alteration in the findings. This is the reason why patient history is important in such studies to ensure the limitations are eliminated.

## 7. Conclusion

When compared to other pain control methods, i.e., SR and EW method, a conclusion can be drawn that FP is an effective method of pain control concerning pain experience during debonding. Teeth in the anterior region of lower and upper arches are more sensitive to pain than the teeth in posterior regions during debonding, irrespective of the pain control methods. A patient's general attitude towards the thought of pain or actual painful condition and patient's sex are important determinants of pain experience during debonding as females tend to report higher pain levels. Most pain scale comparisons for accuracy have been done in the medical sphere and it would be immensely useful to carry out a comparative analysis of different pain scales to determine the most accurate scale for nonthreatening cross-situational orthodontic procedures.

## Figures and Tables

**Table 1:** Descriptive statistics about patient distribution.

		Finger pressure	Stress relief	Elastomeric wafer	Test value	*P* value
Gender	Male	4^a^	6^a^	4^a^		
	Female	16^a^	14^a^	16^a^	0.37^*∗*^	0.83

Age		18.80	19.90	19.90	1.20^	0.54

^*∗*^denotes a significant difference (*P* < 0.05).

**Table 2:** Descriptive statistics for PCS scores.

PCS score	Finger pressure	Stress relief	Elastomeric wafer	Chi square	*P* value
Rumination	5^a^	5^a^	8^b^	18.20	≤0.001^*∗*^ (s)
Magnification	4^a^	3^b^	4^a^	10.40	0.02^*∗*^ (s)
Helplessness	4^b^	5^a^	6^a^	6.63	0.04^*∗*^ (s)
Total	14	12	18	19.39	≤0.001^*∗*^ (s)

^*∗*^(*P* < 0.05) and statistically significant. Post hoc test was conducted to pinpoint a difference within the groups. Different alphabets express that there is a statistically significant difference: EW has a high score in the rumination group compared to FP and SR. SR has the lowest score compared to FP and EW in terms of magnification. FP has the lowest score compared to SR and EW in terms of helplessness.

**Table 3:** Descriptive statistics for VAS scores.

VAS score	Finger pressure	Stress relief	Elastomeric wafer	Chi square	*P* value
Total VAS	6.59	7.49	12.23	6.34	0.043^*∗*^ (S)
Upper VAS	4.21	6.25	10.51	7.08	0.039^*∗*^ (S)
Upper right	3.47	6.53	8.41	5.08	0.08^*∗*^ (NS)
Upper left	4.95	5.97	12.60	9.03	0.037^*∗*^ (S)
Upper anterior	4.30	7.05	12.58	8.45	0.041^*∗*^ (S)
Upper posterior	4.67	6.75	9.34	8.32	0.032^*∗*^ (S)
Lower VAS	8.67	8.86	13.99	7.83	0.048^*∗*^ (S)
Lower right	8.64	8.33	13.34	2.24	0.02^*∗*^ (S)
Lower left	8.71	9.38	14.64	9.03	0.037^*∗*^ (S)
Lower anterior	10.56	9.26	14.23	10.23	0.033^*∗*^ (S)
Lower posterior	7.57	9.54	14.40	8.54	0.029^*∗*^ (S)

^*∗*^(*P* < 0.05) and statistically significant.

**Table 4:** Regression analysis for VAS with treatment group.

	Mean	Lower	Upper	*β*	*R* Square	Adjusted *R*^2^	*P* value
Total VAS	8.77	7.31	10.62	0.27	0.14	0.14	0.03
Upper VAS	6.99	5.96	7.34	0.36	0.13	0.13	0.04
Upper right	6.14	5.30	7.04	0.34	0.16	0.12	0.06
Upper left	7.84	7.75	8.12	0.37	0.14	0.14	0.04
Upper anterior	7.97	7.44	8.06	0.41	0.17	0.13	0.02
Upper posterior	7.34	6.45	8.46	0.28	0.18	0.14	0.03
Lower VAS	10.51	6.31	14.71	0.36	0.14	0.14	0.04
Lower right	10.10	5.94	14.26	0.34	0.12	0.12	0.06
Lower left	10.91	6.57	15.25	0.37	0.17	0.15	0.04
Lower anterior	11.35	7.01	15.69	0.43	0.14	0.13	0.02
Lower posterior	10.86	7.31	14.64	0.36	0.16	0.14	0.03

**Table 5:** Effect of gender on VAS and PCS scores.

Scale	Median (range)		*P* value
	Female	Male	
*VAS scores*			
Overall	7.05 (2.87–43.87)	6.60 (1.35–27.31)	0.55
Upper total	5.00(1.75–39.38)	5.75 (1.00–14.40)	0.75
Upper right	4.75 (1.25–31.50)	4.50 (1.00–14.60)	0.86
Upper left	5.40 (2.25–47.25)	6.40 (1.00–16.00)	0.78
Upper anterior	5.50 (1.83–45.33)	6.17 (0.83–17.00)	0.94
Upper posterior	5.10 (1.70–31.50)	5.9 (1.00–13.40)	0.53
Lower total	8.60 (4.10–48.38)	6.3 (1.00–42.63)	0.04 (S)^*∗*^
Lower right	9.80 (3.60–46.75)	5.6 (1.00–42.75)	0.02 (S)^*∗*^
Lower left	7.40 (2.75–50.00)	6.5 (1.00–42.75)	0.46
Lower anterior	10.67 (4.50–41.67)	7.6 (0.50–49.17)	0.04 (S)^*∗*^
Lower posterior	8.80 (2.50–43.50)	5.8 (1.00–41.50)	0.03 (S)^*∗*^

*PCS scores*			
Rumination	6 (5–8)	6 (3–10)	0.15
Magnification	4 (1–5)	3 (1–7)	0.97
Helplessness	5 (4–7)	5 (3–7)	0.13
Total	14 (12–20)	14 (7–20)	0.50

^*∗*^(*P* < 0.05) and statistically significant.

**Table 6:** Correlations of component and total scores of PCS with VAS scores.

	Rumination			Magnification			Helplessness			Total PCS		
	M	F	T	M	F	T	M	F	T	M	F	T
Overall	0.37^*∗*^	0.38^*∗*^	0.38^*∗*^	0.09	0.10	0.09	0.36^*∗*^	0.34^*∗*^	0.34^*∗*^	0.37^*∗*^	0.38^*∗*^	0.37^*∗*^
Upper total	0.31^*∗*^	0.33^*∗*^	0.31^*∗*^	0.08	0.08	0.08	0.29^*∗*^	0.27^*∗*^	0.27^*∗*^	0.30^*∗*^	0.30^*∗*^	0.30^*∗*^
Upper right	0.27^*∗*^	0.23^*∗*^	0.25^*∗*^	0.02	0.04	0.02	0.30^*∗*^	0.30^*∗*^	0.30^*∗*^	0.24	0.22	0.22
Upper left	0.34^*∗*^	0.34^*∗*^	0.34^*∗*^	0.17	0.16	0.15	0.23	0.26	0.23^*∗*^	0.27^*∗*^	0.37^*∗*^	0.34^*∗*^
Upper anterior	0.33^*∗*^	0.31^*∗*^	0.31^*∗*^	0.10	0.09	0.09	0.26^*∗*^	0.29^*∗*^	0.27^*∗*^	0.31^*∗*^	0.30^*∗*^	0.30^*∗*^
Upper posterior	0.31^*∗*^	0.28^*∗*^	0.30^*∗*^	0.10	0.12	0.10	0.25^*∗*^	0.31^*∗*^	0.29^*∗*^	0.25	0.29	0.27
Lower total	0.40^*∗*^	0.42^*∗*^	0.42^*∗*^	0.64	0.56	0.61	0.35^*∗*^	0.31^*∗*^	0.32^*∗*^	0.38^*∗*^	0.37^*∗*^	0.37^*∗*^
Lower right	0.44^*∗*^	0.42^*∗*^	0.42^*∗*^	0.69	0.71	0.71	0.34^*∗*^	0.33^*∗*^	0.33^*∗*^	0.33^*∗*^	0.39^*∗*^	0.38^*∗*^
Lower left	0.43^*∗*^	0.39^*∗*^	0.40^*∗*^	0.55	0.50	0.50	0.36^*∗*^	0.30^*∗*^	0.32^*∗*^	0.37^*∗*^	0.36^*∗*^	0.36^*∗*^
Lower anterior	0.41^*∗*^	0.42^*∗*^	0.42^*∗*^	0.74	0.69	0.70	0.29^*∗*^	0.27^*∗*^	0.27^*∗*^	0.30^*∗*^	0.39^*∗*^	0.36^*∗*^
Lower posterior	0.40^*∗*^	0.43^*∗*^	0.43^*∗*^	0.63	0.62	0.62	0.32^*∗*^	0.34^*∗*^	0.32^*∗*^	0.35^*∗*^	0.39^*∗*^	0.38^*∗*^

^*∗*^(*P* < 0.05) and statistically significant.

## Data Availability

All data generated or analyzed during this study are included within this article.
